# The chickpea root rot complex in Saskatchewan, Canada- detection of emerging pathogens and their relative pathogenicity

**DOI:** 10.3389/fpls.2023.1117788

**Published:** 2023-02-06

**Authors:** Cheryl Armstrong-Cho, Nimllash Thangam Sivachandra Kumar, Ramanpreet Kaur, Sabine Banniza

**Affiliations:** Crop Development Centre, University of Saskatchewan, Saskatoon, SK, Canada

**Keywords:** disease survey, fusarium avenaceum, fusarium redolens, verticillium dahliae, berkeleyomyces (thielaviopsis) basicola

## Abstract

Chickpea fields in Saskatchewan, one of the three Canadian prairie provinces, have suffered from major health issues since 2019, but no definitive cause has been determined. Field surveys were conducted in Saskatchewan in 2020 and 2021 in order to develop a better understanding of root rot pathogens associated with chickpea. Root samples were analyzed for the presence of 11 potential chickpea root rot pathogens using end-point PCR. *Fusarium redolens*, *F. solani* and *F. avenaceum* were the most prevalent pathogen species detected in both survey years. The cause of Fusarium wilt in chickpea, *F. oxysporum* f. sp. *ciceris*, was not detected in either year, nor were *Phytophthora* spp. and *Verticillium albo-atrum*. *Berkeleyomyces* sp. was detected in one field in each year, and *Verticillium dahliae* was detected in several fields sampled in 2021. These two pathogens have not been reported previously on chickpea in Saskatchewan. The prevalence of *Fusarium* species obtained from 2021 root isolations was similar to that determined by molecular tests, with frequent isolation of *F. redolens*, *F. oxysporum*, *F. avenaceum* and *F. solani*. A series of indoor pathogenicity testing compared root disease severity caused by a selection of 16 isolates of six *Fusarium* species and single isolates of *V. dahliae*, *Berkeleyomyces* sp. and *Macrophomina phaseolina*. Results showed that select isolates of *F. avenaceum* were the most aggressive of the *Fusarium* isolates on chickpea. Despite relatively low inoculum density, a highly aggressive isolate of *F. avenaceum* caused severe stunting and more root rot symptoms than single isolates of *V. dahliae*, *Berkeleyomyces* sp. and *M. phaseolina* under the test conditions.

## Introduction

Chickpea (*Cicer arietinum* L.) is an important pulse crop grown in Saskatchewan, one of the three Canadian prairie provinces, accounting for 78% of Canadian chickpea production in 2021 (Government of Saskatchewan, 2021). The majority of chickpeas grown in Saskatchewan are the kabuli type, which have a thin, colorless seed coat, making them susceptible to attack by a variety of soil-borne pathogens. Seed treatment with fungicide, particularly to control damping off caused by *Pythium* spp., is a routine part of disease management programs in this region. In addition to *Pythium* and *Rhizoctonia* spp., several *Fusarium* spp. can cause economically damaging root rot to chickpea worldwide (Haware, 1998; Infantino et al., 2006; Chen et al., 2011). The intensification of pulse production on the prairies has resulted in increased prominence of root rots, including those caused by *Fusarium* spp. The wide-spread occurrence of Aphanomyces root rot in this region makes chickpea an attractive alternative to lentil and pea in crop rotations due to their high partial resistance (Moussart et al., 2008). However, it has been suspected that root rots caused by other pathogens have been increasing. To date, the spectrum of root-rot pathogens prevalent in the chickpea cropping system, particularly *Fusarium* spp. and their potential for causing significant disease, are unknown, while this has been well studied in pea and lentil during the last decade.

*Fusarium avenaceum* (Fr.) Sacc. and *F. solani* (Mart.) Sacc. (syn. *Neocosmospora solani* (Mart.) L. Lombard & Crous) are the predominant *Fusarium* species in the root rot complex attacking pea and lentil (Esmaeili Taheri et al., 2017, Chatterton et al., 2019). Both of these species are known to impact emergence and cause moderate to severe symptoms on chickpea roots (Kraft, 1969; Westerlund et al., 1974; Safarieskandari et al., 2021). Besides root rot, *F. avenaceum* also contributes to the development of Fusarium head blight in cereal crops (Tekauz et al., 2000; Xue et al., 2004, Tekauz et al., 2004). In a recent study, isolates of *Fusarium redolens* Wollenw., *F. culmorum* (Wm.G. Sm.) Sacc., *F. sporotrichioides* Sherb. (now *Fusarium chlamydosporum Wollenw. & Reinking*), *F. oxysporum* Schltdl. and *F. equiseti* (Corda) Sacc. obtained from diseased chickpea samples were all confirmed to be pathogenic on chickpea (Zhou et al., 2021). The most aggressive isolates on chickpea were of *F. culmorum* and *F. chlamydosporum*, but there were also isolates of these species with low aggressiveness. In addition to root rot, Fusarium wilt of chickpea, caused by *F. oxysporum* f. sp. *ciceris* Matuo & K. Sato, can cause devastating losses in many chickpea growing areas, including most of those found in Asia, Africa, southern Europe, and the Americas (Jiménez-Díaz et al., 2015; Jha et al., 2020). This pathogen has not been reported in Canada.

In addition to *Fusarium* spp., several other chickpea root pathogens have been reported around the globe. *Berkeleyomyces basicola* (Berk. & Broome) W.J. Nel, Z.W. de Beer, T.A. Duong & M.J. Wingf (formerly *Thielaviopsis basicola* Berk. & Broome) which causes black streak root rot, was reported from chickpea roots in eastern Washington in 1985 (Bowden et al., 1985). *Macrophomina phaseolina* (Tassi) Goid. (dry root rot) and *Verticillium albo-atrum* Reinke & Berthold (Verticillium wilt) have been reported in California (Erwin, 1958; Westerlund et al., 1974). These three pathogens have not been reported from chickpea grown in the North American Prairies. Verticillium wilt of canola caused by *V. dahliae* Kleb. was recently reported on the prairies (Hwang et al., 2017) but has not been observed in Canadian chickpea (Chen et al., 2011). Similarly, although *Phytophthora medicaginis* E.M. Hansen & D.P. Maxwell, (Phytophthora root rot) is a pathogen of alfalfa fields in North America, Phytophthora root rot is not common in alfalfa in Saskatchewan (Bill Biligetu, Crop Development Centre/Dept. of Plant Sciences, University of Saskatchewan, personal communication) and it has not been recorded from chickpea crops in the USA or Canada.

## Materials and methods

### Field survey

Commercial chickpea fields in Saskatchewan were surveyed in June and July of 2020 and 2021. The scope of the survey included 41 rural municipalities with 42 commercial chickpea fields and one research location with chickpea breeder plots in 2020. Rural municipalities where chickpea root rot symptoms were most prevalent in 2020 were chosen for sampling in 2021, which included 19 commercial chickpea fields in 14 rural municipalities. Above-ground disease symptoms were recorded for five plants at each of ten locations in each field in 2020, and for five plants each at five locations in each field in 2021. Disease scoring was performed according to a 1-5 qualitative scale adapted from Infantino et al. (2006), in which 1 = no symptoms, 2 = slight yellowing of lower leaves, 3 = yellowing of the lower leaves up to the 3rd or 4th node and some stunting, 4 = necrosis of at least half or more of the plant with some stunting, 5 = entire plant dead or nearly so. Roots were collected at five locations in each field and submitted to the University of Saskatchewan Pulse Crop Pathology Laboratory for further analysis. Due to laboratory access restrictions during the COVID-19 pandemic, roots submitted in 2020 were immediately frozen and not assessed for visual root symptoms. In 2021, root rot symptoms were rated on dry root samples using the 1-7 scale described by Safarieskandari et al. (2021): 1 = no symptoms, 2 = 0.1–0.2 cm, small reddish brown lesions at seed attachment area, 3 = coalescing of localized tap root lesions approximately 180° around the tap root with lesions from 0.5 to 1 cm, 4 = lesions extending and completely encircling the tap root (1–2 cm), 5 = increasingly discoloured and extended tap root lesions (2–4 cm), 6 = lesions encircling the tap root extending over 4 cm and 7 = tap root completely brown/black.

### Molecular detection of potential root pathogens

A total of 208 root samples collected in 2020 and 93 samples collected in 2021 were freeze-dried (FreeZone 6, Labconco Corp., Kansas City MO USA) and ground for DNA extraction. Grinding was performed using custom designed tubes (high strength polycarbonate, Metalshapes Manufacturing, Saskatoon) containing a 1.7 cm diameter stainless steel ball placed in a homogenizer (2010 Geno/GrinderTM, SPEX Sample Prep, Metuchen, NJ USA) at 1400 rpm for 5 min (2020) or at 1000 rpm for 2 min (2021). Ground tissue (approx. 10 mg) was transferred to microcentrifuge tubes along with a 0.6 cm diameter ceramic bead for a second grinding step (1400 rpm for 1 min). Extraction of DNA from ground tissue was conducted using a DNeasy Plant Mini Kit (Qiagen, Hilden, Germany) according to the manufacturer’s instructions, with elution volume reduced to 60 µL. Concentration and quality of DNA (260/280 nm and 260/230 nm ratios) were assessed using a NanoDrop spectrophotometer (Thermo Scientific, Waltham MA, USA), and DNA concentration was diluted to 20 ng µL^-1^.

For molecular detection of pathogens, primer sets specific to various root rot pathogens were selected based on their prior use in the scientific literature and successful amplification of DNA of their particular target. Cross-reaction of primers with other closely related pathogens of relevance to the project was evaluated to determine the possibility of false positive results. The primers chosen for pathogen detection and their respective positive controls are listed in **Table 1**. Of the 12 primer sets, five were originally designed with a central TaqMan probe, but were used as conventional primers without the probe. The IPC primer set, which detects ascomycete fungi (Kulik, 2011), was redesigned using Primer 3 Plus software (Untergasser et al., 2012) and renamed IPC9 (**Table 1**). This primer set combines part of the probe with the reverse primer and uses an upstream forward primer.

**Table 1:** Primers used for pathogen detection in DNA samples derived from chickpea roots collected in Saskatchewan in 2020 and 2021.

Target species	Forward Primer	Reverse Primer	Target Locus	Reference	Positive Control
General ascomycete fungi	IPC9f ACTTTTAACAACGGATCTCTTGGT	IPC9r CAATGTGCGTTCAAAGATTCGATG	5.8S rDNA	modified from Kulik, 2011	F56**
*Fusarium redolens*	RedF*	RedR*	EF1a	Willsey et al., 2018	FR05**
*Fusarium solani*	SolF*	SolR*	EF1a	Willsey et al., 2018	DAOMC 193418
*Fusarium avenaceum*	AveF*	AveR*	EF1a	Willsey et al., 2018	F56**
*Fusarium chlamydosporum*	AF330109CF	AF330109CR	TRI13	Demeke et al., 2005	F47**
*Fusarium culmorum*	Fc01F	Fc01R	RAPD derived	Nicholson et al., 1998	C1 (S. Chatterton)
*Fusarium oxysporum f.* sp. *ciceris*	Foc0-12f	Foc0-12r	SCAR marker	Jiménez-Gasco and Jiménez-Díaz, 2003	SB12 (W. Chen)
*Verticillium dahliae*	Df	Dr	ITS	Inderbitzin et al., 2013	DAOMC 250722
*Verticillium albo-atrum*	Aaf	AaTr	ACT	Inderbitzin et al., 2013	DAOMC 216604
*Phytophthora* spp.	18Ph2F	5.8S-1R	ITS1	Scibetta et al., 2012	DAOMC BR 610
*Berkeleyomyces* sp.	Tb1*	Tb2*	ITS	Huang and Kang, 2010	DAOMC 187829
*Macrophomina phaseolina*	MpKFI*	MpKRI*	ITS	Babu et al., 2007	CBS 205.47

*primers were used without the aid of TaqMan probes.

**identification done in-house based on Ef1a sequence identity with sequences in Fusarium ID and NCBI databases.

Detection of pathogens was accomplished through end-point PCR of 20 µL reactions consisting of 1X buffer, 2.5 mM MgCl_2_, 125 µM dNTP mixture, 0.1 µM of each primer, 1 U of Taq DNA Polymerase (Invitrogen recombinant), and 40 ng of genomic DNA. In order to avoid non-specific bands with primers designed for *F. culmorum*, the MgCl_2_ concentration was reduced to 2 mM. Cycler conditions were 95°C for 4 min, followed by 34 cycles of 95°C for 30 s, 60°C for 30 s and 72°C for 30 s, finished with a final extension at 72°C for 7 min. Amplicons were run on 1.5% agarose gel containing GelRed^®^ (Biotium, Freemont CA USA) for 1 h at 120 v and visualized using a ChemiDoc (Bio-Rad, Hercules CA USA).

Detection of previously unreported pathogens by PCR (*Berkeleyomyces basicola, Verticillium dahliae* and *Macrophomina phaseolina*) was confirmed by sequencing the band produced by their respective species-specific primers (**Table 1**). DNA from excised bands was extracted using a monarch gel extraction kit (New England Biolabs, Ipswich MA USA) and samples were sent for sequencing (Eurofins Genomics, Louisville KY USA). Sequence data were used to construct a trimmed consensus contig (DNA Baser, Heracle Biosoft, Arges, Romania) which was compared with sequences in the NCBI Genbank database (Altschul et al., 1990).

### Pathogen isolation and identification

Frozen tissues in 2020 and air-dried root tissues in 2021 were used for pathogen isolation. Root segments were surface sterilized for 2 min in 10% bleach solution, rinsed in sterile deionized water and placed on potato dextrose agar (PDA) medium for 7-10 days. Fungal colonies were selected based on colony morphology to exclude common saprophytes. Colonies were purified by transferring single germinated conidia to fresh medium. Culture plugs were stored in milk-glycerol solution at -80°C.

Mycelia were produced for DNA extraction by growing purified isolates in liquid medium (1 g NH_4_H_2_PO_4_ [Millipore Sigma], 0.2 g KCl [Fisher Chemical], 0.2 g MgSO_4_ x 7 H_2_O [Millipore Sigma], 10 g D-glucose [Fisher Chemical], 5 g yeast extract [Fisher Chemical], 0.01 g ZnSO_4_ x 7 H_2_O [Millipore Sigma], 0.005 g CuSO_4_ x 5 H_2_O [Millipore Sigma], 1 L distilled water) on a rotary shaker for 2-4 days, filtered to remove media and freeze dried. Freeze-dried tissues were pulverized inside microcentrifuge tubes containing a 0.6 cm diameter ceramic bead using a custom-made paint can shaker at full speed for 1 min. Extraction, quantification and dilution of DNA were conducted as described above.

Soil was collected from the research field from which *Berkeleyomyces* sp. had been detected by PCR in 2020. Desi chickpea seedlings were grown in this field soil and the root tissue was used for pathogen isolation as described above. Examination of endoconidia and chlamydospore morphology (Nel et al., 2018) was used to select *Berkeleyomyces*-like colonies. Identification of a *Berkeleyomyces sp.* isolate was confirmed with species-specific primers (Huang and Kang, 2010), and sequence data was generated and analyzed as described above.

Since morphological identification of *Fusarium* species is often unreliable, species-specific primers were also used to identify isolates of common *Fusarium* species by end-point PCR (**Table 1**). Reactions were processed as described above, except that 1.5 mM MgCl_2_ and 1 ng µL^-1^ of genomic DNA were used. For *Fusarium* isolates with inconclusive identification using selective primers, the TEF1 locus was sequenced after amplification with primers EF1 and EF2 (Geiser et al., 2004). Extraction of PCR amplicons, sequencing and data analysis were performed as described above. In addition to using the NCBI database, results were submitted to the online Fusarium identification tool (fusarium.mycobank.org, CBS-KNAW Fungal Biodiversity Centre).

### Pathogenicity testing

A series of three pathogenicity tests were conducted for comparisons among isolates obtained from field surveys and those obtained from culture collections. All experiments were conducted in controlled environment chambers (Conviron model GR-48, Winnipeg, Canada) with 25°C daytime, 10°C night temperature and a 16 h photoperiod. Plants were grown in 10 cm diameter pots of peat-based medium (Sunshine mix #4, Sun Gro Horticulture, Agawam, MA USA or ProMix-BX-general purpose soil mix, Premier Tech Horticulture, Rivière-du-Loup, QC Canada). Cultures were grown on PDA for 5 to 7 days under incandescent lighting at room temperature. To prepare spore suspension of *F. culmorum*, PDA cultures were rinsed with deionized water and filtered through miracloth (Calbiochem, San Diego, CA USA). For spore production of all other *Fusarium* species, two plugs cut from the growing edge of the colony on PDA were added to a 250 mL flask containing 100 mL of carboxymethyl cellulose (CMC) medium and incubated under light for 4-5 days at 23°C on a shaker at 150 rpm (Foroud et al., 2012). After filtering through miracloth, conidia in liquid cultures were harvested by centrifugation for 5 min at 3400 rpm, followed by two washes with deionized water. Following re-suspension in deionized water, the resulting suspension was adjusted to 1 x 10^4^ spores mL^-1^. This suspension was added to moist growth medium at a rate of 3 x 10^6^ conidia per kg (300 mL of 1 x 10^4^ conidia mL^-1^) prior to planting. Ten days after seeding, fresh conidia suspensions were prepared as described above and adjusted to 1 x 10^3^ conidia mL^-1^. Aliquots of 5 mL were pipetted to the base of each seedling, henceforth referred to as drenching. Water was pipetted to the base of seedlings in non-inoculated controls. Seedling emergence was recorded 10 days after planting. Root rot severity was assessed 3 weeks after planting by assessing disease development on the hypocotyl. All experiments had four replicates arranged in a randomized complete block design and were conducted twice.

Experiment 1 included three chickpea survey isolates each of *F. redolens* (FR06, FR08, FR10) and *F. solani* (FSL01, FSL03, FSL04) as well as one isolate of *F. avenaceum* (Fav7). Two additional local isolates of *F. avenaceum*, Fav3 from pea and Fav 5 from lentil, which had previously been used for germplasm screening of various other pulse crops, were included. Seeds of CDC Orkney (kabuli) and CDC Sunset (desi) were surface sterilized in 10% bleach for 2 min and rinsed twice with deionized water prior to seeding in inoculated potting mix and further processed as described above. Disease severity was assessed on a 0-5 scale (modified from Coyne et al., 2019), where 0 indicated no disease symptoms, 1 indicated small hypocotyl lesions, 2 indicated lesions coalescing around epicotyls and hypocotyls, 3 indicated lesions starting to spread into the root system with some root tips infected, 4 indicated epicotyl, hypocotyl and root system almost completely infected and 5 indicated a completely infected root and dead plant.

Experiment 2 compared the most aggressive *F. avenaceum* (Fav3, Fav5), *F. solani* (FSL04) and *F. redolens* (FR06) isolates evaluated in the first experiment to an isolate of *F. oxysporum* f. sp. *ciceris* from Washington state (race 1 isolate SB12, W. Chen, USDA ARS, Dept. of Crop and Soil Sciences, and Plant Pathology, Washington State University) and 6 other local *Fusarium* isolates. These included four *F. culmorum* from chickpea (FC04, FC05, FC06, FC07), one *F. inflexum* from chickpea (Fi01) and one *F. inflexum* from lentil (Fi02). Inoculation of soil at planting and 10 days later was performed as described above. Seeds of kabuli chickpea cultivar CDC Leader were planted as described above, but without surface sterilization. Plant height was measured on 3-week-old plants prior to removing plants from pots for disease assessment. Disease assessment was performed using a 0-10 incremental scale (0 = no symptoms, 1 = 1 to 10% of root tissue affected, 2 = 11 to 20% of root tissues affected, and so on, to 10 = 91 to 100% of root tissues affected) to indicate the degree of damage to the hypocotyl region.

In Experiment 3, disease severity caused by a local, highly aggressive *F. avenaceum* (Fav5) was compared to disease caused by single isolates of *V. dahliae* (DAOMC 250722, from soil, Ontario), *M. phaseolina* (CBS 205.47, from common bean, Italy), and a local isolate of *Berkeleyomyces* sp. from chickpea (TB02). The kabuli cultivar CDC Leader was planted after seeds were surface sterilized as described above. For *F. avenaceum*, *Berkeleyomyces* sp. and *M. phaseolina*, soil incorporation of inoculum was followed by drenching 10 days after seeding as previously described, with method modifications to suit the biology of each pathogen, including inoculum preparation, concentration, and, for *V. dahliae*, delivery method. Inoculum preparation and concentrations for *F. avenaceum* followed the standard protocol described above. Cultures of *Berkeleyomyces* sp. were grown on PDA and incubated for 10 days at room temperature under continuous incandescent lighting. Chlamydospores and endoconidia (which were the majority of spores) were harvested by flooding the Petri dishes with sterile tap water, scraping with a sterile glass slide, and filtering the suspension through miracloth. Based on prior research, the spore suspension was adjusted to 1 x 10^4^ spores mL^-1^ for soil incorporation and drenching (Tabachnik et al., 1979). Cultures of *M. phaseolina* were grown on oatmeal agar medium incubated at room temperature for 10 days under continuous incandescent lighting. Mycelia were harvested by flooding the Petri dishes with sterile distilled water and scraping the culture surface with a sterile glass slide. The mycelia were homogenized in a blender and adjusted to 3 x 10^4^ mycelia fragments per mL for soil incorporation and for drenching (modified from Cota-Barreras et al., 2022). Cultures of *V. dahliae* were prepared on PDA and in CMC medium for spore production as described above. Based on prior research, spore suspensions were adjusted to 3 x 10^7^ spores mL^-1^, and a seedling root soaking method was used for inoculation (Jiménez-Fernández et al., 2016). Seedlings were grown in medium horticultural vermiculite (Perlite Canada Inc., Lachine QC Canada) and removed from their pots 7 days after seeding. Seedlings were soaked in spore suspension for 15 min and transplanted into non-inoculated ProMix-BX-general purpose soil mix (Premier Tech Horticulture, Rivière-du-Loup, QC Canada). Spore suspension of 3 x 10^7^ spores mL^-1^ was used for drenching 10 days after transplanting. Non-inoculated controls were maintained for each isolate (species) treatment in order to capture any effect of seedling dipping and transplanting or the soil incorporation method to help determine relative differences in disease severity. Plant height was measured prior to removing plants from pots for disease assessment. Disease assessment was performed using the 0-10 incremental scale to indicate the degree of damage to the hypocotyl region.

### Statistical analysis

All analyses were conducted using SAS software version 9.4 (SAS Institute, Cary NC, USA). Pooling of experimental runs was performed after ensuring that there was no statistical effect of experimental run. Analysis of ordinal disease rating data from pathogenicity experiment 1 was performed following conversion to rank using the rank procedure. The mid-ranks (r), the default in the rank procedure, were then used in the mixed procedure to calculate the nonparametric test statistics and their significance levels (P-values). Genotype, treatment and genotype by treatment were considered fixed effects. The Wald-type statistic (WTS) was computed using the Chi-squared test. The anovaf option in the mixed procedure was used to generate the calculation of the ANOVA-type statistic (ATS), and the repeated statement was used to specify properties of the variances within experimental units (Shah and Madden, 2004).

For pathogenicity experiments 2 and 3 that had percentage disease data, normality of errors were evaluated with the Shapiro–Wilk test and homogeneity of variance with the Levene’s test before being modelled with the mixed model procedure. Replicate nested in experimental runs and experimental run were considered random effects whereas isolates were considered fixed effects. Heterogeneous variances were modeled with the repeated statement as required. Means were separated based on Fisher’s least significant difference at P = 0.05.

## Results

### Field survey

Spring moisture was adequate in the chickpea growing area of Saskatchewan in 2020, but moisture was limited in spring 2021. Summer conditions in both years were characterized by below average rainfall along with hot temperatures and drying winds. Mean disease severity assessments of above-ground symptoms (yellowing, stunting, necrosis) on a 1 to 5 rating scale ranged from 1.1 to 4.1 in 2020 and from 2.2 to 4.4 in 2021 when averaged for each field (**Tables S1**, **S2**). The median score for all fields assessed was 1.8 in 2020, indicating slight yellowing on above-ground plant parts in many fields at most assessment locations in each field. It is noteworthy that even in those fields with low average disease scores, 39 of the 43 fields had locations that were rated with scores of 3 and 4, indicating the possibility of serious root rot foci in the majority of fields. Fields in 15 rural municipalities had average ratings of 3 and higher indicating moderate to severe yellowing and stunting, and in four RMs, dead plants were observed (rating score of 5). Rural municipalities where the most severe root rot symptoms were observed in 2020 were selected for sampling in 2021. Of the 19 fields surveyed in 2021, 16 fields had maximum ratings of 4 or 5, indicating that severe symptoms and/or dead plants were observed in most fields. Heat and drought stress likely contributed to these symptoms, as may have other unknown factors.

Assessment of root rot severity in 2021 was performed on dry roots, which made fine features of lesions more difficult to observe. Mean severity of root rot symptoms ranged from 1.6, indicating only very small lesions, to 6.0, indicating extensive lesion development on the taproot. The overall mean root rot severity for the 19 fields was 2.9, which demonstrates that root rot damage was significant despite dry growing conditions. Severe root rot (rating of 4 to 7) was observed in root samples from seven of the 19 fields (**Table S2**).

### Molecular detection of potential root pathogens

Primer testing demonstrated that cross-reactions among the species involved in this study were only observed for *F. culmorum* primers Fc01F/R (Nicholson et al., 1998). This primer set resulted in cross reaction with several other species at 2.5 mM MgCl_2_, including *V. albo-atrum*, *F. avenaceum*, *F. solani*, *F. redolens*, and *F. chlamydosporum*. Reduction of MgCl_2_ to 2 mM eliminated most cross-reactions so that only a faint band persisted with *F. redolens*. Although this primer set has been cited extensively in the literature, it has only been used in the context of cereal pathology, and thus its specificity was not tested against a full spectrum of *Fusarium* species and other fungi.

**Figure 1:**
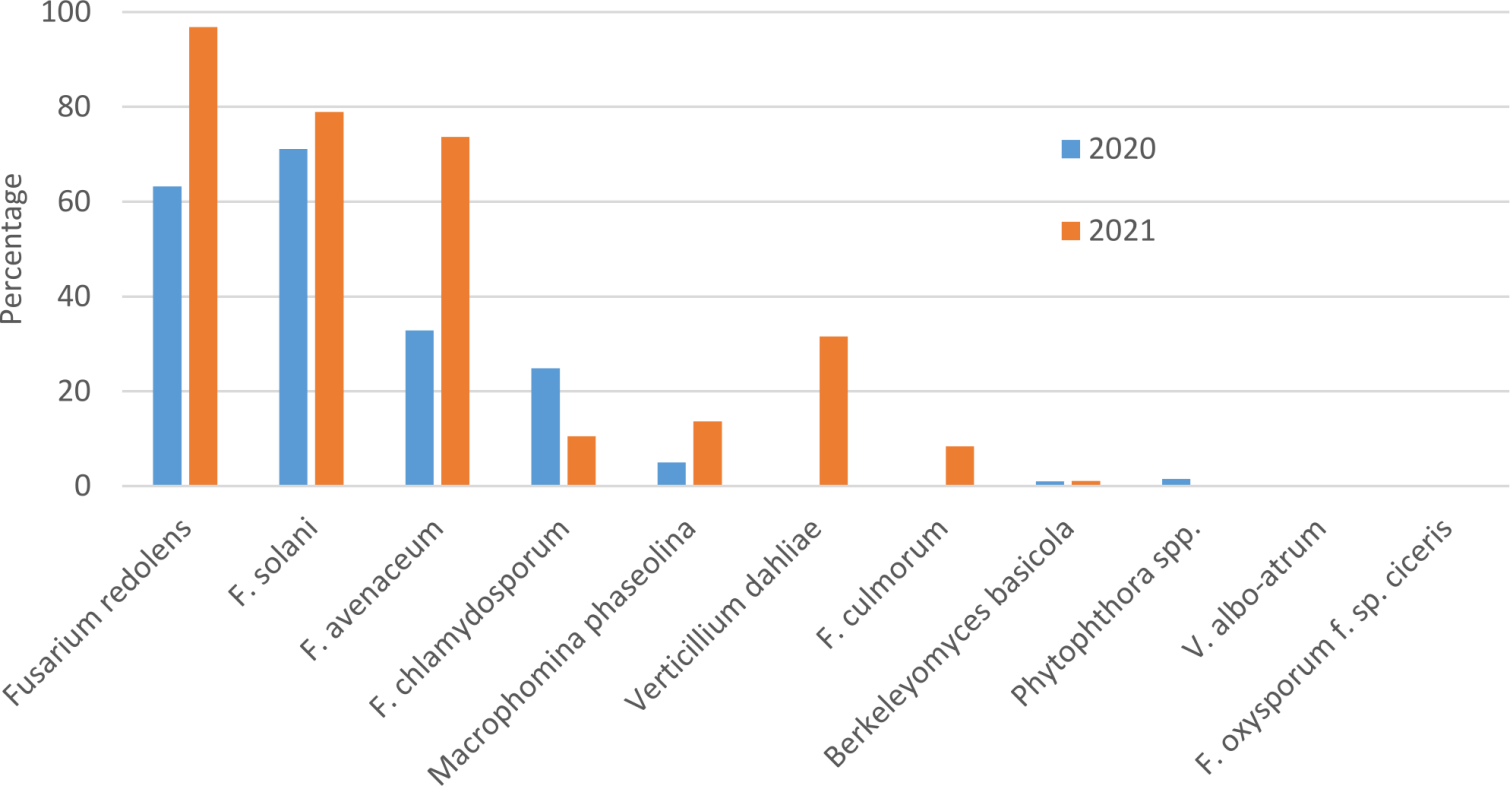
Percentage of chickpea root samples collected in Saskatchewan in 2020 and 2021 in which potential chickpea root pathogens were detected by end-point PCR.

In both survey years, *Fusarium solani* and *F. redolens* were the most prevalent pathogens detected in root samples, but *F. solani* was the most frequently detected pathogen in 2020 samples, whereas *F. redolens* was most frequent in 2021 samples. *Fusarium avenaceum* was also frequently detected in 2021 samples (73%), whereas it was only present in 33% of samples in 2020. *Fusarium chlamydosporum* was also detected in both years at relatively low frequency. *Fusarium culmorum* was not detected in any of the 2020 samples, and at a relatively low frequency (9%) in 2021 samples (**Figure 1**).

Amplicons of the expected size were obtained with primers designed for detection of *Macrophomina phaseolina* in 5% of 2020 and 14% of 2021 samples, but attempts to sequence these bands were unsuccessful, suggesting that amplification was non-specific. *Berkeleyomyces basicola* was detected in two 2020 samples originating from the University of Saskatchewan research farm in Saskatoon, and from 1 sample from a commercial farm in 2021. Although not detected in 2020 samples, *Verticillium dahliae* was detected in 33% of 2021 samples (**Figure 1**). Bands amplified with the *Berkeleyomyces* sp. and *V. dahlia*e-specific primers (**Table 1**) were sequenced to confirm species identity. A 312 bp consensus sequence generated from the *Berkeleyomyces* sp. band (OQ183437) had 100% coverage and 100% identity with NCBI sequences for a reference strain of *B. basicola* (MF952429) and the type strain of *B. rouxiae* (MF952412.1). Identity with both of these reference strains at an rDNA locus is not surprising, as these organisms were only recently split into two species, *B. basicola* and *B. rouxiae* W.J. Nel, Z.W. de Beer, T.A. Duong & M.J. Wingf. (Nel et al., 2018). Bands obtained from two root samples using the *V. dahliae* primers generated 498 bp (OQ183438) and 508 bp (OQ183439) sequences, which had 99.48% and 97.22% identity with NCBI sequences for the type specimen of *V. dahliae* (NR_126124.1) with 77% coverage. Higher coverage (99%) was observed for *V. dahliae* accession HE972025.1, with 99.6% identity for the 498 bp contig and 98.02% identity for the 508 bp contig. Both *Berkeleyomyces* sp. and *V. dahliae* have not been reported previously from chickpea in Saskatchewan.

*Fusarium oxysporum* f. sp. *ciceris* and *V. albo-atrum* were not detected in any samples. Detection of members of the *Phytophthora* genus were rare, with 1% of samples in 2020 but zero in 2021 (**Figure 1**).

### Pathogen isolation and identification

Pathogen isolation from chickpea root tissues resulted in the purification of 7 *Fusarium* spp. isolates in 2020, and 52 *Fusarium* spp. isolates in 2021. Of the 59 *Fusarium* spp. isolates, 58 were identified using a combination of species-specific primers and sequencing at the Ef1a locus. Sequencing was performed for 20 *Fusarium* spp. isolates, including all putative *F. oxysporum* (OQ181356 to OQ181375). Seventeen isolates were *Fusarium redolens*, 13 F*. oxysporum*, ten *F. avenaceum*, seven *F. solani*, five *F. culmorum*, three *F. caucasicum* Letov, one *F. incarnatum-equiseti* complex, one *F. acuminatum* or *F. tricinctum* complex and one *F. toxicum* L. Lombard & J.W. Xia. The identity of one isolate remained undetermined. No *F. oxysporum* f. sp. *ciceris* isolates were obtained.

One *Berkeleyomyces* isolate was obtained from chickpea seedlings grown in field soil collected from a research site in 2020. The amplicon obtained for DNA of this isolate using species-specific primers (Huang and Kang, 2010) was of expected size and matched the amplicon size obtained with a culture collection isolate (DAOMC 187829). A 318 bp consensus sequence generated from the band had 100% coverage and 100% identity with NCBI sequences for a reference strain *of B. basicola* (MF952429.1) and the type strain of *B. rouxiae* (MF952412.1). Due changes in fungal taxonomy that occurred after the publication of the primer set and inability to resolve species based on the ITS sequence, we refer to this isolate as *Berkeleyomyces* sp.

### Pathogenicity testing

**Figure 2:**
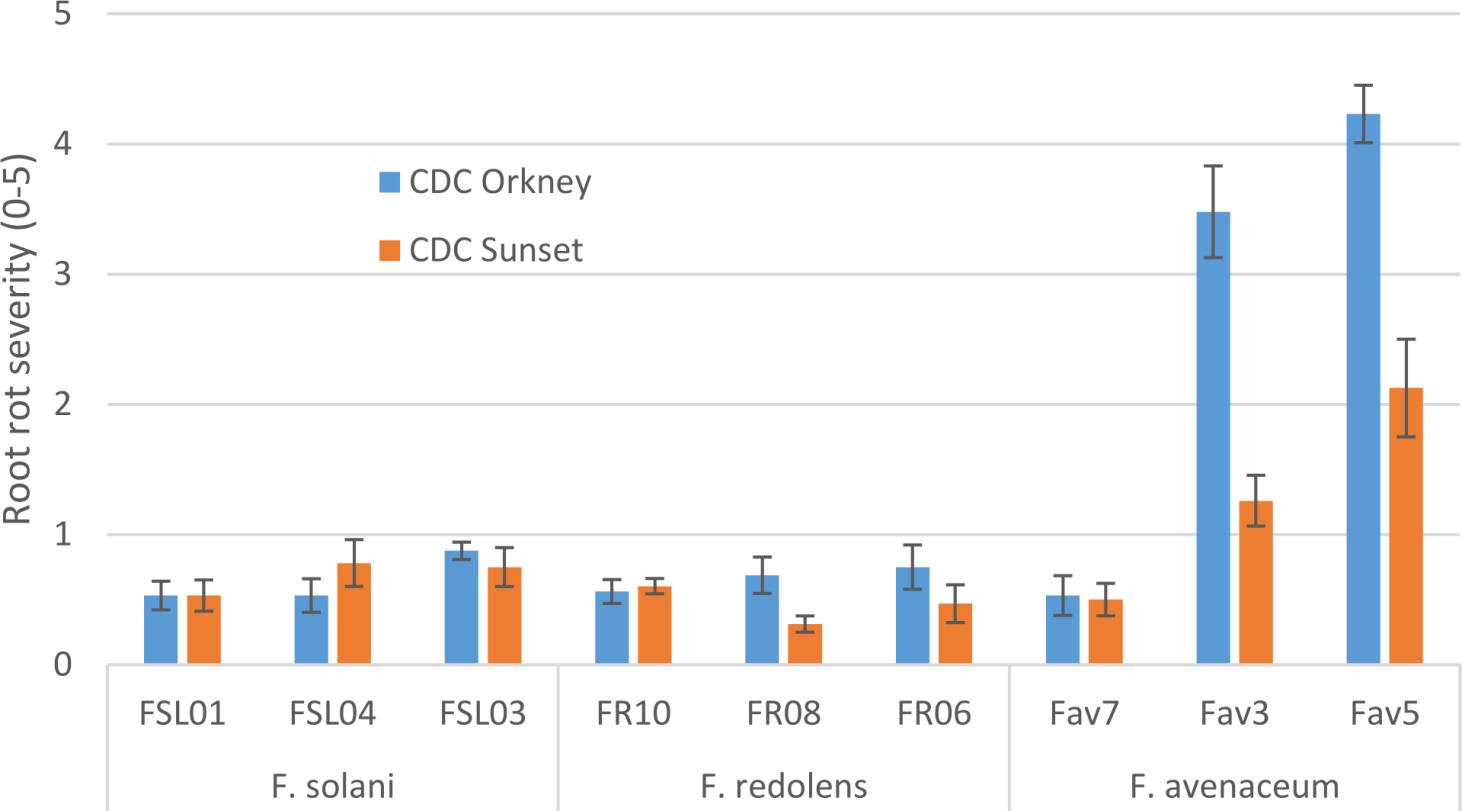
Root rot severity (0-5 scale) caused by three isolates each of three *Fusarium* species on 3-week-old plants of CDC Orkney kabuli chickpea and CDC Sunset desi chickpea under controlled conditions. Inoculum was incorporated into soil at planting and applied by soil drenching 10 days after planting.

In growth chamber tests comparing three isolates each of *F. redolens*, *F. avenaceum* and *F. solani* (Experiment 1), chickpea cultivars and isolates both had significant effects on root rot severity (P ≤ 0.014), but their interaction was not significant (P = 0.18). Disease severity was highest for two isolates of *F. avenaceum* (Fav 3, Fav5) on both the desi and kabuli cultivar tested. The remaining isolates caused only limited disease, with mean ratings of less than 1 on both cultivars (**Figure 2**). The two most aggressive isolates of *F. avenaceum* (Fav 3, Fav5) originating from pea and lentil caused a 16 to 22% reduction in emergence of the kabuli cultivar (data not shown). These isolates, as well as *F. redolens* (FR06) and *F. solani* (FSL04), were chosen to be included in Experiment 2.

**Figure 3:**
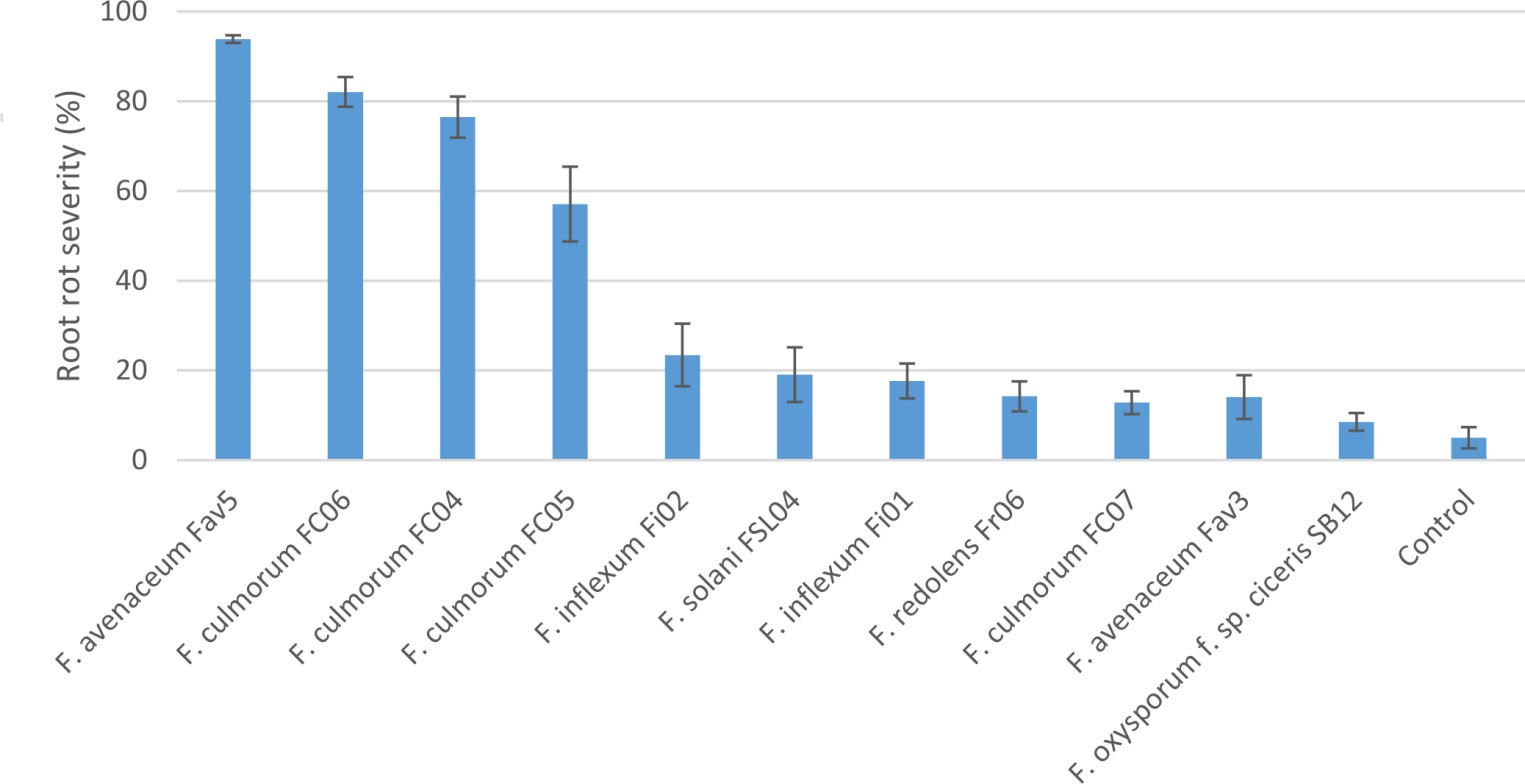
Root rot severity (%) caused by nine isolates of six *Fusarium* species on 3-week-old plants of CDC Leader kabuli chickpea under controlled conditions. Inoculum was incorporated into soil at planting and applied by soil drenching 10 days after planting.

**Figure 4:**
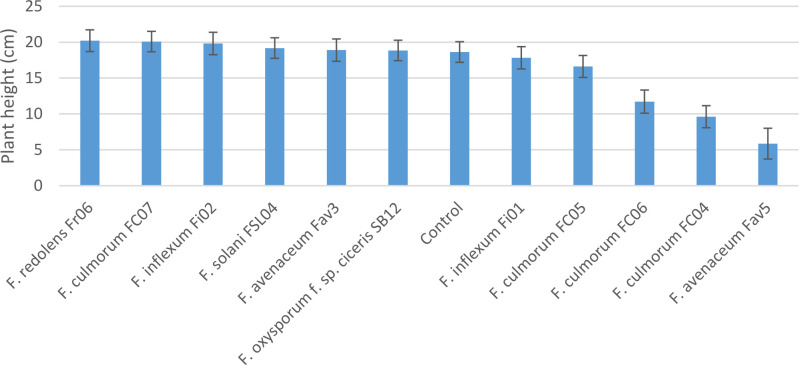
Height (cm) of 3-week-old CDC Leader kabuli chickpea inoculated with nine isolates of six *Fusarium* species under controlled conditions. Inoculum was incorporated into soil at planting and applied by soil drenching 10 days after planting.

Experiment 2 included nine selected isolates of six different *Fusarium* species. None of the isolates had a significant effect on emergence of kabuli cultivar CDC Leader (P = 0.075) but isolate significantly impacted plant height and root rot severity (P<0.0001 for both). Single isolates of *F. solani*, *F. culmorum*, *F. redolens* and both *F. inflexum* isolates caused low disease severity (<24%, **Figure 3**). Disease severity observed for the Fav3 isolate of *F. avenaceum* and an isolate of *F. oxysporum* f. sp. *ciceris* did not differ from that observed in the non-inoculated controls. This result was not unexpected for *F. oxysporum* f. sp. *ciceris*, given that this organism causes vascular wilting rather than root rot symptoms, but this isolate also failed to cause any height reduction of CDC Leader within the 21-day time frame of the experiment (**Figure 4**). Three of the *F. culmorum* isolates caused moderate to severe root rot symptoms, ranging from 57% to 82% severity. This was significantly less than the 94% disease severity caused by *F. avenaceum* isolate Fav5 (**Figure 3**). These same four isolates (*F. culmorum* FC04, FC05, FC06, and *F. avenaceum* Fav5) were the only ones that caused a significant height reduction relative to non-inoculated CDC Leader plants (**Figure 4**).

**Figure 5:**
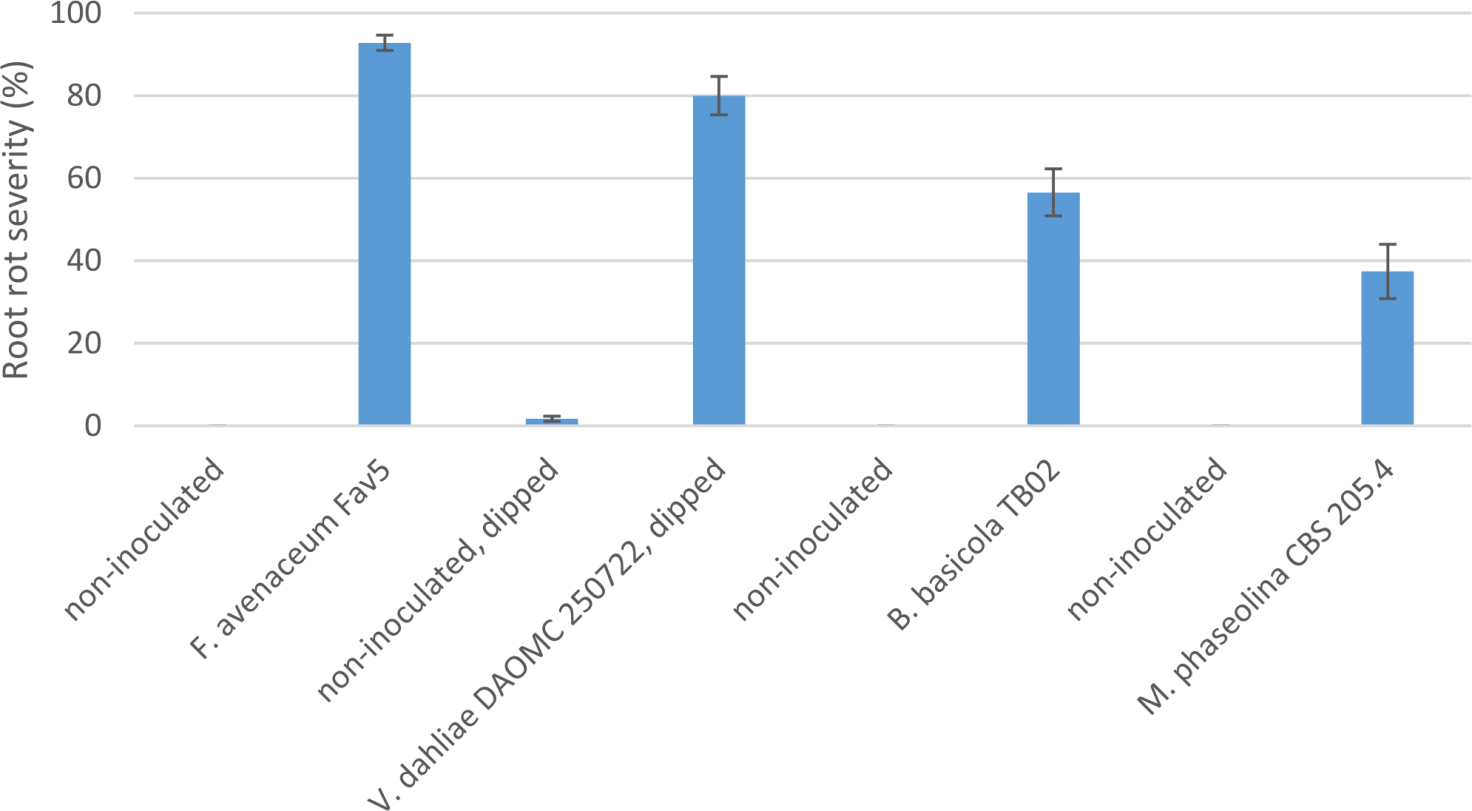
Root rot severity (%) caused by four pathogens on 3-week-old plants of CDC Leader kabuli chickpea under controlled conditions. Inoculum was incorporated into soil at planting or applied as a seedling dip, and applied by soil drenching 10 days after seeding or transplanting.

**Figure 6:**
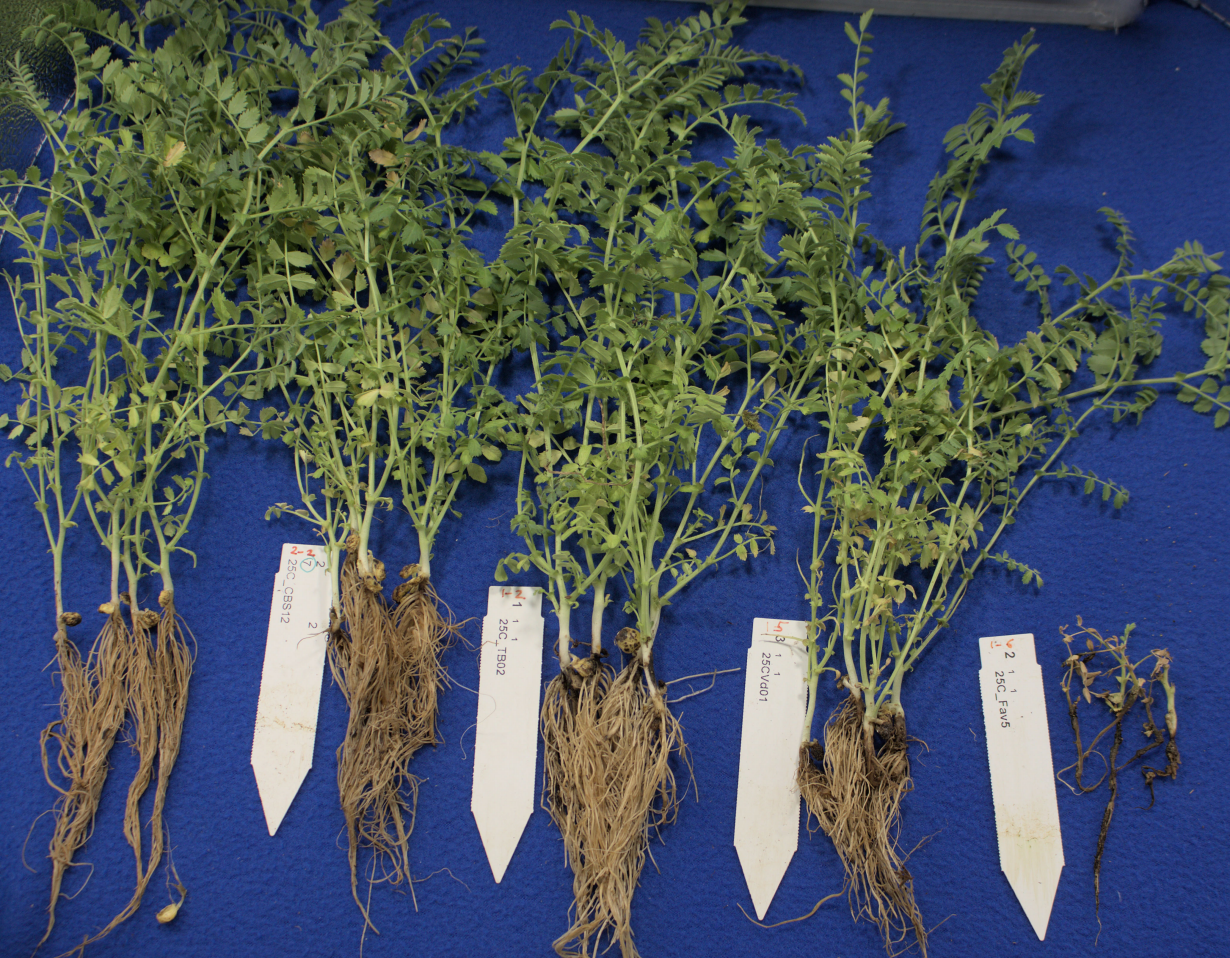
Root rot symptoms caused by four pathogens on 3-week-old plants CDC Leader kabuli chickpea under controlled conditions. Inoculum was incorporated into soil at planting or applied as a seedling dip, and applied by soil drenching 10 days after seeding or transplanting. From left to right: non-inoculated control, *Macrophomina phaseolina*, *Berkeleyomyces basicola*, *Verticillium dahliae*, *Fusarium avenaceum*.

Comparison of pathogenicity of single isolates of *M. phaseolina*, *Berkeleyomyces* sp., and *V. dahliae* to a local, highly aggressive isolate of *F. avenaceum* (Fav5) used in the two prior experiments in Experiment 3, revealed no significant effect of the isolates on emergence (P = 0.84), but isolate impacted height (P < 0.0001) and root rot severity (P < 0.0001). Plants in the non-inoculated controls accompanying soil-incorporated inoculum treatments showed no root rot, and non-inoculated plants that had been removed from their pots and dipped in deionized water showed only trace root discoloration (<2%, **Figure 5**). In the combined data analysis, the only isolate to significantly impact plant height was *F. avenaceum* Fav5, which resulted in 79% height reduction relative to non-inoculated control plants (data not shown). *Verticillium dahliae* significantly reduced plant height in one of the two experimental runs, but this effect was not statistically supported by means comparisons with combined data. Disease severity differed significantly for the single isolates of the four pathogens, with *F. avenaceum* causing the most damage to roots, followed by *V. dahliae*, *Berkeleyomyces* sp. and *M. phaseolina* under the test conditions (**Figure 5**). Infection by *F. avenaceum* resulted in totally collapsed brown hypocotyls and a small, brown tap root. Plants were severely stunted, dying or dead (**Figure 6**). Plants infected with *V. dahliae* developed brown lesions that encircled the hypocotyl. Disease development was more severe in experimental run 2, where *V. dahliae* infection affected the whole hypocotyl, epicotyl and tap root, and caused significant height reduction (P = 0.009). The impact on height observed in experimental run 2 was not statistically supported in pooled data. Symptoms caused by *Berkeleyomyces* sp. included black lesions on the hypocotyl that were generally not more than 1 cm in length and did not encircle the hypocotyl. Black lesions were also observed on hypocotyls of *M. phaseolina*-infected plants, but these were discrete lesions of limited size (**Figure 6**). Infection by *Berkeleyomyces* sp. and *M. phaseolina* did not impact plant height (data not shown).

## Discussion

The increasing prominence of root rot diseases in pulse crops is an important issue facing agricultural systems on the Canadian prairies. Along with *Aphanomyces euteiches*, *Fusarium avenaceum* and *F. solani* are major pathogens of pea and lentil in this region (Chatterton et al., 2019). Chickpea cultivars grown in this region are susceptible to Fusarium root rot caused by *F. avenaceum* (S. Banniza, unpublished). During surveys of chickpea fields during 2020 and 2021, the typical pattern of patchy root rot development was observed under the prevailing hot, dry conditions. The 2021 survey focused upon regions where wilting, discoloration and/or stunting of chickpea were observed in 2020, which lead to a higher proportion of symptomatic plants and/or roots observed in 2021 as compared to 2020. No obvious geographical localization of individual root pathogens was detected in the chickpea production area surveyed. In both years, *F. redolens*, *F. avenaceum* and *F. solani* DNA detections were the most prevalent, with a prominent increase in *F. avenaceum* and *F. redolens* detection frequency in 2021 over that in 2020. The use of *M. phaseolina* species-specific primers gave misleading results due to non-specific amplification. Despite using a standard technique implemented successfully by other researchers (Mayék-Pérez et al., 2001; Cota-Barreras et al., 2022), this pathogen was never observed in PDA isolation plates, suggesting that *M. phaseolina* is not a current threat to chickpea production in Saskatchewan. Previously unreported pathogens *V. dahliae* and *Berkeleyomyces* sp. were detected in chickpea root tissue using molecular techniques, but whereas *Berkeleyomyces* sp. was isolated from chickpea roots, *V. dahliae* was never observed in isolation plates. While it is possible that isolation of *V. dahliae* would have benefitted from the use of semi-selective medium, other researchers have successfully used PDA for isolation of *V. dahliae* (Jabnoun-Khiareddine et al., 2010; Ashraf et al., 2012). Despite molecular detection of *V. dahliae* in 12 field samples, the pathogen could not be reliably isolated using these standard techniques. It is possible that the use of dry root tissue for isolation also did not favor *Verticillium* recovery. The impact of *V. dahliae* infection of chickpea under field conditions warrants further investigation, particularly given the wide host range of this pathogen and its potential for impact on canola, which is widely grown on the Canadian prairies (Hwang et al., 2017).

Pathogenicity studies involving the three most prevalent *Fusarium* species detected in surveys showed that three *F. redolens* isolates and three *F. solani* isolates caused only very mild symptoms on chickpea roots. This is supported by a previous study using two *F. redolens* isolates from durum which caused low to moderate disease on pea, desi chickpea and durum, with one of these isolates causing more disease on pea than on desi chickpea or durum (Esmaeili Taheri et al., 2011). Of the three *F. avenaceum* isolates in the current study, two caused severe disease on CDC Orkney kabuli chickpea, whereas only one of these, an isolate obtained from lentil, caused severe disease on CDC Leader kabuli chickpea. The lone chickpea isolate chosen for pathogenicity testing caused only minor symptoms. Some variation in the aggressiveness of 19 F*. avenaceum* isolates was previously observed on pea and chickpea (Safarieskandari et al., 2021). When compared to isolates of *F. solani*, *F. culmorum*, *F. redolens*, *F. oxysporum* and *F. acuminatum*, six of the 19 isolates of *F. avenaceum* were aggressive enough to kill pea plants, and one isolate caused only moderate symptoms on pea. A mixture of three of these *F. avenaceum* isolates with high aggressiveness on pea were inoculated onto two kabuli and two desi cultivars. Reduced emergence of one desi and one kabuli cultivar following inoculation using a seed soaking method was observed, and CDC Leader, a kabuli cultivar also used in the current study, did not emerge. Moderate root rot severity was reported for the chickpea cultivars that successfully emerged. The large variation in aggressiveness among *F. avenaceum* isolates reported by Safarieskandari et al. (2021) and observed in the current study suggests that further work is needed to investigate whether host origin relates to isolate aggressiveness and host preference. Given the potential for *F. avenaceum* to impact pulse and cereal crops, further research on this topic could be used to improve disease management strategies.

Three of the four *F. culmorum* isolates tested under controlled conditions caused moderate to severe root rot severity and significant height reduction in inoculated chickpea plants. Research comparing *Fusarium* sp. isolates from pea showed that isolates of *F. culmorum* caused root rot symptoms equivalent to that caused by the most aggressive *F. avenaceum* isolates on pea seedlings (Safarieskandari et al., 2021). During chickpea root rot surveys conducted during two dry, hot seasons, *F. culmorum* was not detected in root tissues collected in 2020, and only at low frequency in 2021. Isolation of *F. culmorum* from root tissue also occurred at low frequency in 2021. Given this pathogen’s potential to cause severe root rot on chickpea and pea indoors where conditions are more moist and temperate, it is worthwhile continuing to learn about its role in the root rot complex of pulse and cereal crops.

Conducting disease survey work provided an opportunity to assess the potential threat of several previously unreported pathogens of chickpea in our region. Given that climate change threatens to modify growing conditions and may thereby shift the importance and composition of pathogen species and populations, creating an inventory of potential pathogens and assessing their relative pathogenicity is one small step toward system resiliency. No isolates of *F. oxysporum* f. sp. *ciceris* were recovered from diseased chickpea roots collected in disease surveys. An isolate of *F. oxysporum* f. sp. *ciceris* race 1 obtained from the US was included in growth chamber testing, where it failed to cause wilting or stunting of CDC Leader. As a pathogen with known impact on chickpea, these results may have been due to unsuitable test conditions, insufficient time for disease development, or resistance of CDC Leader to race 1 Fusarium wilt. Three additional root rot pathogens of chickpea with international significance, *Berkeleyomyces* sp., *V. dahliae* and *M. phaseolina*, were compared to a local, highly aggressive isolate of *F. avenaceum*. As these pathogens all have distinct biology and infection strategies, a direct, subjective comparison is somewhat difficult to attain. In addition, since these pathogens have not previously been reported in our region, we only had access to a single local isolate of *Berkeleyomyces* sp. and had to rely on single isolates of *V. dahliae* and *M. phaseolina* from culture collections. Despite these limitations, pathogenicity testing conducted using methods tailored to each pathogen provided some interesting insight. Although the inoculum density for the *F. avenaceum* isolate was the lowest of all four pathogens, this organism caused the most severe damage to chickpea seedlings under the test conditions. Within 20 days, chickpea inoculated with *F. avenaceum* Fav5 were dead or dying, and those inoculated with *V. dahliae* in one experimental run exhibited stunting. Chickpea seedlings inoculated with *Berkeleyomyces* sp. and *M. phaseolina* still appeared healthy above-ground but had hypocotyl lesions developing. Continued vigilance and assessment of the impact of emerging chickpea pathogens *V. dahliae* and *Berkeleyomyces* sp. under field conditions is recommended.

## Data availability statement

The datasets presented in this study can be found in online repositories. The names of the repository/repositories and accession number(s) can be found below: https://www.ncbi.nlm.nih.gov/genbank/, OQ181356 OQ181357 OQ181358 OQ181359 OQ181360 OQ181361 OQ181362 OQ181363 OQ181364 OQ181365 OQ181366 OQ181367 OQ181368 OQ181369 OQ181370 OQ181371 OQ181372 OQ181373 OQ181374 OQ181375 OQ183436 OQ183437 OQ183438 OQ183439.

## Author contributions

All authors listed have made a substantial, direct, and intellectual contribution to the work and approved it for publication. Cheryl Armstrong-Cho and Nimllash Thangam Sivachandra Kumar have contributed equally to this work and share first authorship.
