# Supplementary material for: The chickpea root rot complex in Saskatchewan, Canada- detection of emerging pathogens and their relative pathogenicity
Source: Front Plant Sci. 2023 Feb 6;14:1117788. doi: 10.3389/fpls.2023.1117788 (PMC9939516; doi:10.3389/fpls.2023.1117788)
Supplement: Supplementary file 1 [file Table_1.docx]

**Table S1.** Symptoms observed in chickpea fields surveyed in Saskatchewan in 2020. Assessments are based on five plants at ten sites in each field.

| **Field Location**  **(Rural Municipality #)** | **Mean Above-Ground**  **Disease Severity (1 - 5)** | **Std Error** |
| --- | --- | --- |
| 189 | 1.1 | 0.05 |
| 51 | 1.2 | 0.06 |
| 229 | 1.3 | 0.07 |
| 134 | 1.4 | 0.09 |
| 108 | 1.5 | 0.08 |
| 131 | 1.5 | 0.08 |
| 74 | 1.5 | 0.10 |
| 72 | 1.5 | 0.11 |
| 261 | 1.6 | 0.09 |
| 67 | 1.6 | 0.11 |
| 171 | 1.6 | 0.07 |
| 10 | 1.7 | 0.10 |
| 71 | 1.7 | 0.10 |
| 99 | 1.7 | 0.10 |
| 73 | 1.7 | 0.08 |
| 135 | 1.7 | 0.09 |
| 135 | 1.8 | 0.07 |
| 259 | 1.8 | 0.09 |
| 138 | 1.8 | 0.09 |
| 103 | 1.8 | 0.20 |
| 111 | 1.8 | 0.07 |
| 231 | 1.8 | 0.10 |
| 19 | 1.8 | 0.07 |
| 226 | 1.9 | 0.12 |
| 107 | 2.0 | 0.08 |
| 38 | 2.0 | 0.13 |
| 39 | 2.1 | 0.12 |
| 104 | 2.1 | 0.08 |
| 231 | 2.2 | 0.10 |
| 163 | 2.2 | 0.11 |
| 100 | 2.2 | 0.20 |
| 191 | 2.3 | 0.12 |
| 102 | 2.4 | 0.12 |
| 161 | 2.4 | 0.09 |
| 162 | 2.5 | 0.13 |
| 9 | 2.7 | 0.08 |
| 43 | 2.8 | 0.09 |
| 12 | 2.9 | 0.08 |
| 167 | 3.0 | 0.02 |
| 7 | 3.0 | 0.05 |
| 130 | 3.2 | 0.11 |
| 344 | 4.0 | 0.22 |
| 8 | 4.1 | 0.16 |
