# Supplementary material for: The chickpea root rot complex in Saskatchewan, Canada- detection of emerging pathogens and their relative pathogenicity
Source: Front Plant Sci. 2023 Feb 6;14:1117788. doi: 10.3389/fpls.2023.1117788 (PMC9939516; doi:10.3389/fpls.2023.1117788)
Supplement: Supplementary file 2 [file Table_2.docx]

**Table S2.** Symptoms observed in chickpea fields surveyed in Saskatchewan in 2021. Assessments are based on five plants at five sites in each field.

| **Field Location**  **(Rural Municipality #)** | **Mean Above-Ground**  **Disease Severity**  **(1 - 5)** | **Std Error**  **Disease Severity** | **Mean Root Rot Severity (1 – 7)** | **Std Error**  **Root Rot Severity** |
| --- | --- | --- | --- | --- |
| 73 | 3.8 | 0.20 | 4.4 | 0.40 |
| 73 | 3.0 | 0.45 | 2.8 | 0.20 |
| 43 | 3.4 | 0.19 | 2.4 | 0.24 |
| 12 | 2.6 | 0.17 | 2.8 | 0.20 |
| 12 | 3.0 | 0.19 | 2.2 | 0.20 |
| 72 | 3.5 | 0.19 | 2.8 | 0.37 |
| 70 | 3.1 | 0.21 | 2.5 | 0.55 |
| 130 | 2.4 | 0.12 | 3.0 | 0.00 |
| 131 | 2.2 | 0.09 | 3.0 | 0.32 |
| 103 | 3.0 | 0.20 | 1.6 | 0.24 |
| 162 | 3.3 | 0.15 | 2.0 | 0.32 |
| 162 | 3.2 | 0.22 | 1.6 | 0.40 |
| 68 | 2.4 | 0.10 | 2.2 | 0.20 |
| 7 | 4.4 | 0.12 | 6.0 | 0.00 |
| 103 | 3.6 | 0.20 | 3.4 | 0.87 |
| 74 | 3.0 | 0.15 | 3.4 | 0.24 |
| 104 | 2.3 | 0.18 | 4.0 | 0.32 |
| 73 | 3.1 | 0.11 | 2.6 | 0.24 |
| 161 | 2.8 | 0.20 | 1.8 | 0.20 |
